# PKCζ facilitates lymphatic metastatic spread of prostate cancer cells in a mice xenograft model

**DOI:** 10.1038/s41388-019-0722-9

**Published:** 2019-01-31

**Authors:** Guangxiang Zang, Yabing Mu, Linlin Gao, Anders Bergh, Marene Landström

**Affiliations:** 1 Department of Medical Biosciences, Building 6M, 2:nd floor, Umeå University, SE 90185 Umeå, Sweden; 2 Present Address: National Institute of Biological Sciences, 7 Science Park Road ZGC Life Science Park, Beijing, 102206 China

**Keywords:** Predictive markers, Target validation

## Abstract

Prostate cancer disseminates primarily into the adjacent lymph nodes, which is related to a poor outcome. Atypical protein kinase C ζ (PKCζ) is highly expressed in aggressive prostate cancer and correlates with Gleason score, clinical stage, and poor prognosis. Here, we report the molecular mechanisms of PKCζ in lymphatic metastasis during prostate cancer progression. Using zinc-finger nuclease technology or PKCζ shRNA lentiviral particles, and orthotopic mouse xenografts, we show that PKCζ-knockout or knockdown from aggressive prostate cancer (PC3 and PC3U) cells, decreasesd tumor growth and lymphatic metastasis in vivo. Intriguingly, PKCζ-knockout or knockdown impaired the activation of AKT, ERK, and NF-κB signaling in prostate cancer cells, thereby impairing the expression of lymphangiogenic factors and macrophage recruitment, resulting in aberrant lymphangiogenesis. Moreover, PKCζ regulated the expression of hyaluronan synthase enzymes, which is important for hyaluronan-mediated lymphatic drainage and tumor dissemination. Thus, PKCζ plays a crucial oncogenic role in the lymphatic metastasis of prostate cancer and is predicted to be a novel therapeutic target for prostate cancer.

## Introduction

Prostate cancer is the most prevalent cancer and second leading cause of cancer death in men. A total of 180,890 new cases of prostate cancer and 26,120 deaths from this disease were reported in the United States in 2016 [1]. Frequently, prostate cancer cells metastasize into adjacent lymph nodes and bone. Lymph node metastasis often indicates a poor prognosis for prostate cancer patients and, if present, has only 20–30% 5-year survival in patients with more than five metastatic lymph nodes and 75–80% 5-year survival in patients with a single metastatic lymph node [2]. Lymph node metastasis also indicates a lower possibility of success for therapeutic treatment of prostate cancer [3]. Therefore, understanding mechanisms behind the lymphatic metastasis of prostate cancer is crucial.

PKCζ, a member of the atypical protein kinase C (aPKC) subfamily, is a calcium- and diacylglycerol-independent serine/threonine-protein kinase [4] that promotes the aggressive phenotype of human prostate cancer. PKCζ expression is highly correlated with Gleason score, clinical stage, and poor survival [5] and enhanced expression of PKCζ has also been reported in breast [6], glioma [7, 8], and pancreatic cancer [9]. Via different signaling pathways, PKCζ plays a crucial role in the regulation of multiple cellular processes. In mitogen-activated protein kinase (MAPK) cascades, PKCζ functions as a MEK1 kinase, activating MEK1 and ERK1 upon stimulation with serum or tumor necrosis factor α (TNFα) [10]. PKCζ also acts as an adaptor protein in the activation of MEK5-ERK5 in the EGF signaling pathway, regulating cell proliferation [11]. Notably, PKCζ is active upstream of the inhibitor of nuclear factor-kappa-B (NF-κB) kinase (IKK) pathway, regulating the activation and translocation of NF-κB dimer p50/p65 into the nucleus. Thus, PKCζ is critically involved in the activation of NF-κB in the Toll-like receptor (TLR) signaling pathway, which is important for regulation of the inflammatory response [12, 13] and cancer progression. PKCζ is also a well-known polarity protein, comprising partition-defective 6 (PAR6) and PAR3, regulating cell polarity, cell migration, and invasion [14].

The tumor microenvironment plays an important role in modulating cancer cell survival, proliferation, and invasion. During tumor progression, a variety of stromal cells in the surrounding environment, such as endothelial cells derived from the bone marrow, are recruited to tumors by cancer cells to construct the blood and lymphatic circulatory system. The inductions of angiogenesis and lymphangiogenesis are crucial for tumor growth and tumor metastatic dissemination. Tumor cells express a number of angiogenic and lymphangiogenic factors to induce the process of angiogenesis and lymphangiogenesis. Importantly, a large number of peripheral blood monocytes from the circulation are also recruited to metastatic sites and differentiate into tumor-associated macrophages (TAMs), promoting lymphangiogenesis, seeding and persistent growth of tumor cells [15–17].

Hyaluronan (HA), a dominant component of the extracellular matrix, is a high molecular weight glycosaminoglycan consisting of a repeated disaccharide. HA is synthesized at the plasma membrane by the hyaluronan synthase enzymes (HASs) and released into the extracellular space. Extracellular HA is subjected to degradation by hyaluronidases (Hyals), and then removed via lymphatic drainage or recycled by resident cells. HA interacts with cell surface receptors, including LYVE-1, CD44, receptor for HA-mediated motility (RHAMM), HA receptor for endocytosis (HARE), intercellular adhesion molecule-1 (ICAM-1), and TLR4, which can initiate several signal transduction pathways [18, 19]. HA plays a critical role in homeostasis, tissue injury and repair, and cancer progression, including the prostate [20]. Lymphatic vessels drain a large number of HA molecules via its receptor LYVE-1 and, by collecting protein-rich fluid and cells, maintain tissue homeostasis, which also facilitates the dissemination of cancer cells [21]. However, little is known about the mechanism whereby prostate cancer cells metastasize via the lymphatics.

In the present study, we investigated the role of PKCζ in lymphatic metastatic spreading during prostate cancer progression. Our results showed that PKCζ-knockout in aggressive human prostate cancer (PC3 and PC3U) cells reduces lymphatic metastasis in vivo. Intriguingly, PKCζ-knockout inhibited the activation of the AKT, ERK, and NF-κB signaling pathways in prostate cancer and lung carcinoma (A549) cells. Moreover, we found that PKCζ regulates the expression of lymphangiogenic factors and the secretion of CCL2 by prostate cancer cells, which is important for the recruitment of monocytes/macrophages and lymphangiogenesis. Furthermore, we found that PKCζ regulates the expression of HASs in prostate cancer cells, which is important for HA production and cancer cell dissemination via lymphatic drainage.

## Results

### Expression of PKCζ and the response of PKCζ inhibitior in prostate cell lines

**Fig. 1:**
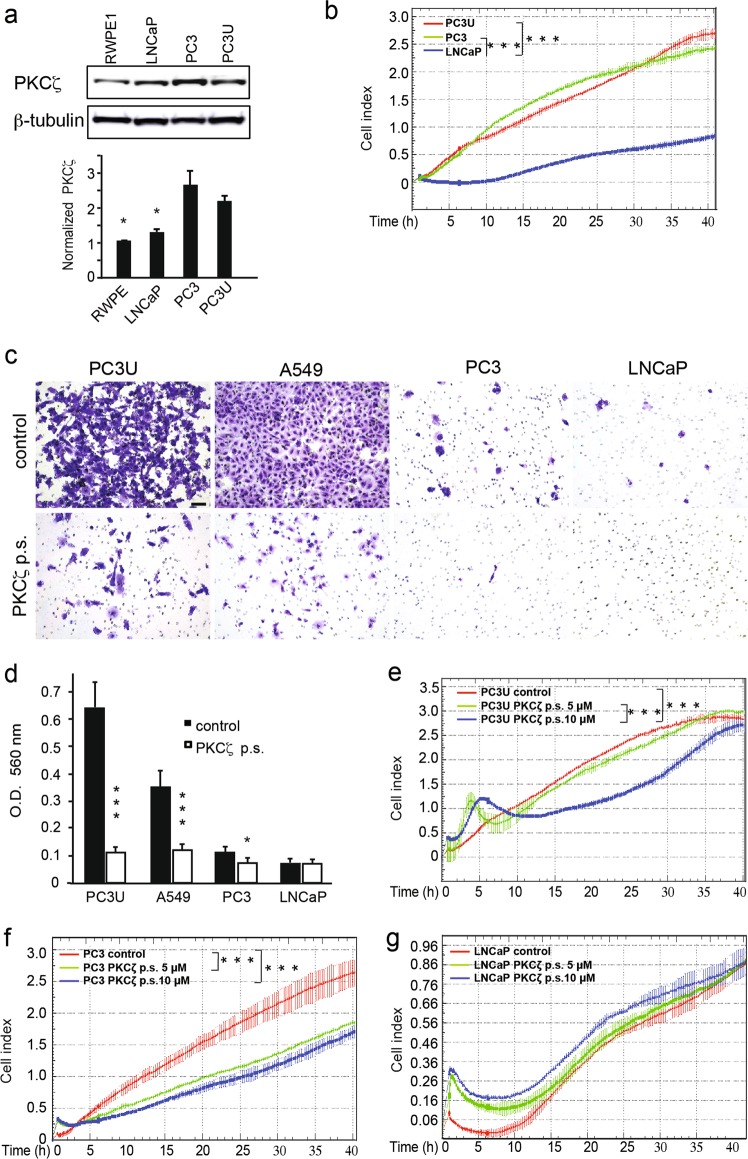
Expression of PKCζ and the response for PKCζ inhibitor in prostate cell lines. **a** Western blot analysis of PKCζ expression in non-malignant human prostate cell line RWPE1 and human prostate cancer cell lines LNCaP, PC3 and PC3U. PKCζ and β-tubulin antibodies were used at 1:1000 dilution. Bar graph represents the mean ± S.D. of 3 independent experiments, * *p* ≤ 0.05, students’ *t* test. **b** Proliferation of PC3U, PC3, and LNCap cells was monitored by a real-time xCelligence-based cell proliferation assay. Representive results from 3 independent experiments are shown as mean ± S.D., ***p ≤ 0.001 as determined by students’ *t* test. **c** Invasion assay for PC3U, A549, PC3, and LNCaP cells treated with PKCζ pseudosubstrate (PKCζ p.s.) or not. Invasive cells were visualized by staining with crystal violet cell stain solution. Scale bar, 50 μm. **d** Mean values for the optical density (OD) of invasive cells. Error bar represents S.D. (*n* = 3 independent experiments, *p ≤ 0.05, ***p ≤ 0.001, one-way ANOVA. **e**–**g** xCelligence-based cell proliferation assay for PC3U, PC3, and LNCap cells treated with PKCζ p.s. or not. Representive results from 3 independent experiments are shown as mean ± S.D. ***p ≤ 0.001 as determined by students’ *t* test

The protein expression levels of PKCζ was investigated by immunoblotting in non-malignant human prostate cell line RWPE1 and human prostate cancer cell lines LNCaP, PC3 and PC3U. The highly aggressive PC3 and PC3U cells expressed higher levels of PKCζ than the non-malignant RWPE1 and LNCaP cells (Fig. 1a). We next analyzed the capability of proliferation for PC3U, PC3, and LNCap cells in real-time in a xCelligence-based cell proliferation assay. The aggressive PC3U and PC3 cells proliferated faster than the less malignant LNCaP cells (Fig. 1b). To investigate if PKCζ expression could affect the invasiveness of several types of prostate cells and the highly aggressive lung cancer A549 cells, we treated cells with the inhibitor of PKCζ, (PKCζ pseudosubstrate; p.s.). We observed that treatment with PKCζ inhibitor significantly reduced invasion of PC3U, A549 and PC3 cells (Fig. 1c, d). We also observed that PC3U cells showed higher invasive capability than PC3 cells (Fig. 1c, d), which was reflected by the higher number of mutated genes in PC3U cells, as found by performing whole genomic sequencing (Supplementary Figure 1A and Fig. 1B). Interestingly, treatment with PKCζ pseudosubstrate, could also inhibit the proliferation of PC3U and PC3 cells, but not LNCaP cells as investigated by a xCelligence proliferation assay (Fig. 1e-g). The proliferation of PC3U and PC3 cells was significantly inhibited by treatment with the PKCζ pseudosubstrate in a dose-dependent manner (5 μM and 10 μM), compared with control (Fig. 1e, f). In contrast, treatment of LNCaP cells with PKCζ pseudosubstrate did not cause inhibition of their proliferative capability (Fig. 1g). From these data, we concluded that the expression of PKCζ was higher in the most aggressive prostate cancer cells (PC3 and PC3U) and correlated with their proliferative and invasive capability, as treatment with PKCζ pseudosubstrate significantly reduced both proliferation and invasion. LNCaP cells with lower levels of PKCζ showed less capability to invade and their proliferation which was not affected by treatment with PKCζ pseuodsubstrate.

### Generation and validation of PKCζ - deficient cancer cells

**Fig. 2:**
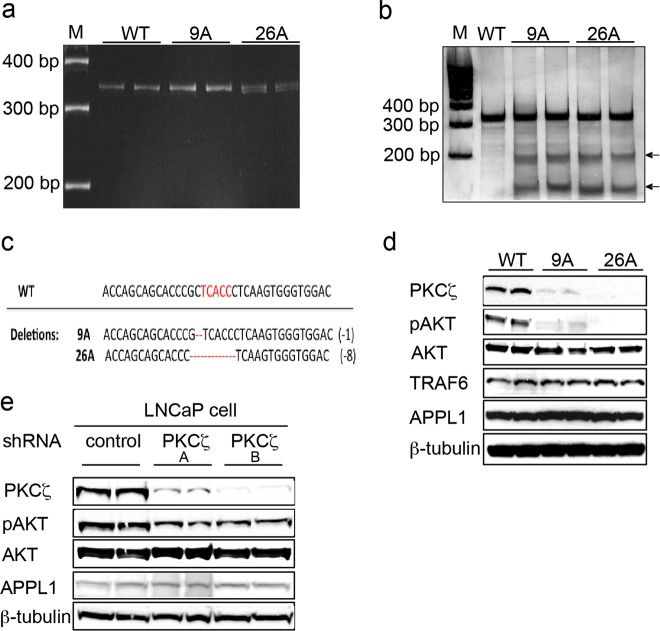
Generation and validation of PKCζ-deficient cancer cell lines. **a** PKCζ gene was knockout in PC3U cells by Zinc Finger Nucleases (ZFN) technology, and PKCζ gene from WT and PKCζ-knockout clones 9A and 26A was amplified by PCR and detected by Single-strand conformation polymorphrism (SSCP) analysis. PKCζ gene mutations were identified by Cel-I (Surveyor nuclease) assay. Arrows indicate the cleavage products generated in Surveyor nuclease assays. **c** Representative sequencing analysis for PKCζ gene deletion induced by ZFN. The red letters represent ZFN-binding sites, and the red dashes represent the deletions in PKCζ gene. **d** Western blot analysis of PKCζ and other proteins expressed in WT PC3U cells, and clones 9A and 26A. **e** LNCaP cells were stably transfected with control shRNA or PKCζ shRNA lentiviral particles (A=10 μl/ml, B= 20 μl/ml), and the total cell lysates were subjected to western blot analysis. Antibodies were used at 1:1000 dilution (**d**, **e**)

The programmable nucleases, such as zinc-finger nucleases (ZFNs), transcription activator-like effector nucleases (TALENs), and clustered regularly interspaced short palindromic repeat/CRISPR-associated protein 9 (CRISPR/Cas9), have been widely used for genetic manipulation in different model systems [22–24]. In our study, CompoZr-ZFNs were used for specific gene disruptions. One pair of knockout ZFN plasmids that specifically target the *PKCζ* gene were purchased from Sigma-Aldrich. Following the protocol, the *PKCζ* gene was knocked out in PC3U cells. Two cell clones were selected: 9A, which has one base-pair deletion, and 26A, which has eight base-pair deletions in the *PKCζ* gene (Fig. 2a-c). The *PKCζ* gene from WT cells, clone 9A, and clone 26A was amplified by PCR and detected by Single-Strand Conformation Polymorphism (SSCP) analysis (Fig. 2a). SURVEYOR mutation detection (CEL-1) assay was performed to detect the double-stranded DNA mismatches in 9A and 26A (Fig. 2b), which indicate deletions in the PKCζ gene in the cell clones. The results were verified by DNA sequencing and whole exome sequencing (Fig. 2c). Immunoblotting showed that the PKCζ protein level was clearly decreased (Fig. 2d), whereas other proteins, such as the ubiquitin ligase TRAF6 and endocytic adaptor protein APPL1, were not affected. Interestingly, activation of AKT was prevented when the PKCζ gene was knocked out in the 9A and 26A cell clones. We investigated also the role for PKCζ on AKT activation in LNCaP cells by knock down of PKCζ by stable transfection of shRNA lentiviral particles, but only a modest effect was found when compared with control shRNA cells when total cell lysates were investigated by immunoblot for p-AKT (Fig. 2e). From these data we concluded that knock down of PKCζ in PC3U cells had a more obvious effects to reduce the activity status of AKT than in LNCaP cells, in line with the higher expression of PKCζ in PC3U cells (as shown in Fig. 1).

### Knockout or knockdown of PKCζ inhibits activation of AKT, ERK and NF-κB signaling

Previous studies have shown that PKCζ is involved in the EGF, TGFβ, and Toll-like receptor (TLR) signaling pathways [10, 12, 13, 25] thus, it was interesting to elucidate the impaired signaling induced by reduced PKCζ expression in PC3U cells.

**Fig. 3:**
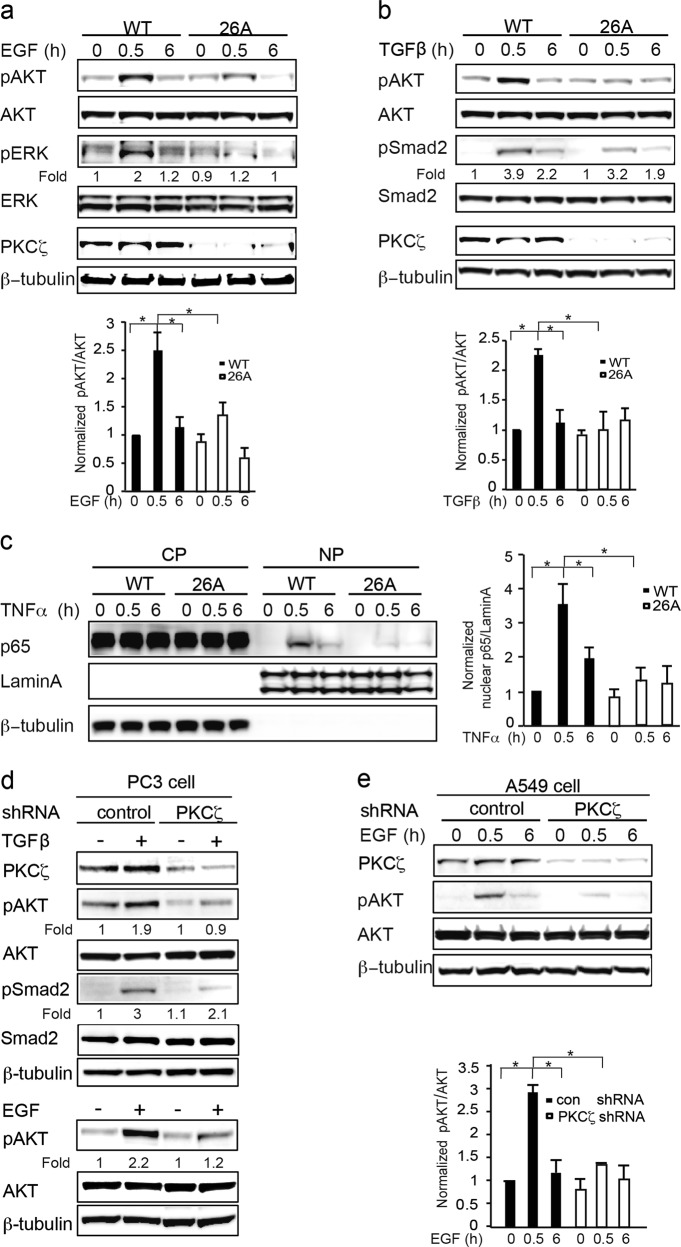
Knockout or knockdown of *PKCζ* inhibited activation of AKT, ERK and NFκB signaling. **a** Western blot analysis of WT and *PKCζ* knockout 26A cells treated with EGF, (**b)** TGFβ, (**c**) TNFα for the indicated time. Bar graph represents the mean ± S.D. of 3 independent experiments, *p ≤ 0.05, students’ *t* test. **d** *PKCζ* was stably knocked down in PC3 cells and (**e**) A549 cells by PKCζ shRNA lentiviral particles, and the total cell lysates were subjected to western blot analysis. Bar graph represents the mean ± S.D. of 3 independent experiments, *p ≤ 0.05, students’ *t* test. Antibodies were used at 1:1000 dilution

First, the effects on the EGF signaling pathway were investigated, showing that the activation of AKT and ERK were inhibited when *PKCζ* was knocked out in 26A cell clones (Fig. 3a). Second, TGFβ is known to activate AKT in WT PC3U cells [25], but activation of AKT was observed to be reduced in 26A cells (Fig. 3b). Third, the activation of NF-κB signaling was investigated by immunoblotting the cytoplasmic and nuclear fractions; p65 was activated and translocated into the nuclei of WT PC3U cells upon TNFα stimulation, whereas less p65 was found in the nuclear fraction from 26A cells (Fig. 3c). Next we extended our study on the effects of PKCζ for selected intracellular signaling pathways to include PC3 cells (Fig. 3d) and A549 cells (Fig. 3e) in which *PKCζ* was stably knocked down by using PKCζ shRNA lentiviral particles. Immunoblotting was used to analyze the role of PKCζ for activation of AKT and Smad2 in response to TGFβ (Fig. 3d) or AKT in response to EGF (Fig. 3e). Upon TNFα stimulation, it was also observed that less p65 was activated and translocated into the nuclei when *PKCζ* was stably knocked down in PC3 cells by immunofluorescence staining (Supplementary Figure 2). These data indicated that knocking out PKCζ in PC3U, PC3 and A549 cells inhibited growth factor induced activation of the AKT, ERK, and NF-κB signaling pathways, which is important for regulation of cell proliferation and invasion in prostate cancer progression.

### Knockout or knockdown of *PKCζ* in cancer cells impairs cell proliferation and invasion

**Fig. 4:**
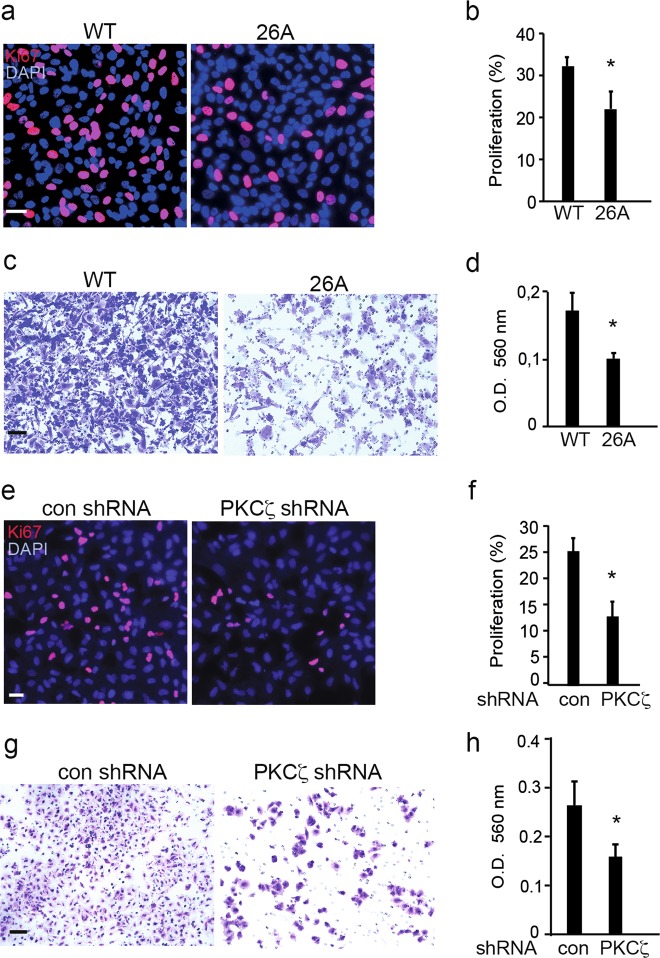
Knockout or knockdown of PKCζ in cancer cells impairs cell proliferation and invasion. **a** Immunofluorescence analysis of Ki-67 in WT PC3U cells and the PKCζ-knockout clones 26A. Scale bar, 50 μm. **b** Quantification of Ki-67-positive cells in total 300 cells. Bar graph represents as mean ± S.D. from 3 independent experiments, *p ≤ 0.05, students’ *t* test. **c** Invasion assay for WT PC3U and the *PKCζ-*knockout clones 26A cells, and the invasive cells were visualized by staining with crystal violet cell stain solution. Scale bar, 50 μm. **d** Mean optical density (O.D.) of invasive cells. Bar graph represents the mean ± S.D. of 3 independent experiments, *p ≤ 0.05, students’ *t* test. **e** Immunofluorescence analysis of Ki-67 in A549 cells stably transfected with control shRNA or PKCζ shRNA lentiviral particles. Scale bar, 50 μm. **f** Quantification of Ki-67-positive cells in more than 300 cells. Bar graph represents the mean ± S.D. of 3 independent experiments, *p ≤ 0.05, students’ *t* test. **g** Invasion assay for A549 cells treated with control shRNA or PKCζ shRNA lentiviral particles, and the invasive cells were visualized by staining with crystal violet cell stain solution. Scale bar, 50 μm. **h** Mean optical density (O.D.) of invasive cells. Bar graph represents the mean and S.D. of 3 independent experiments, *p ≤ 0.05, students’ *t* test. Antibodies for Ki-67 were used at 1:500 dilution (**a**, **e**)

Since we observed that knock down of *PKCζ* affected growth factor induced activation of several key intracellular signaling pathways, we investigated next the biological relevance of these observation by analyzing their eventual effects on proliferation and invasion. Reduced proliferation as measured by Ki-67, and invasion was observed in the PKCζ-knockout clones 26A compared with WT PC3U cells (Fig. 4a-d). Knock down of PKCζ in A549 cells by PKCζ shRNA lentiviral particles in comparision with control shRNA, impaired also their capability to proliferate and be invasive (Fig. 4e-h). From these data we conclude that expression of PKCζ is important for growth factor induced proliferation and invasion of prostate and lung cancer cells.

### Knockout or knockdown of *PKCζ* reduces tumor growth and lymphatic metastasis in an orthotopic xenograft model

**Fig. 5:**
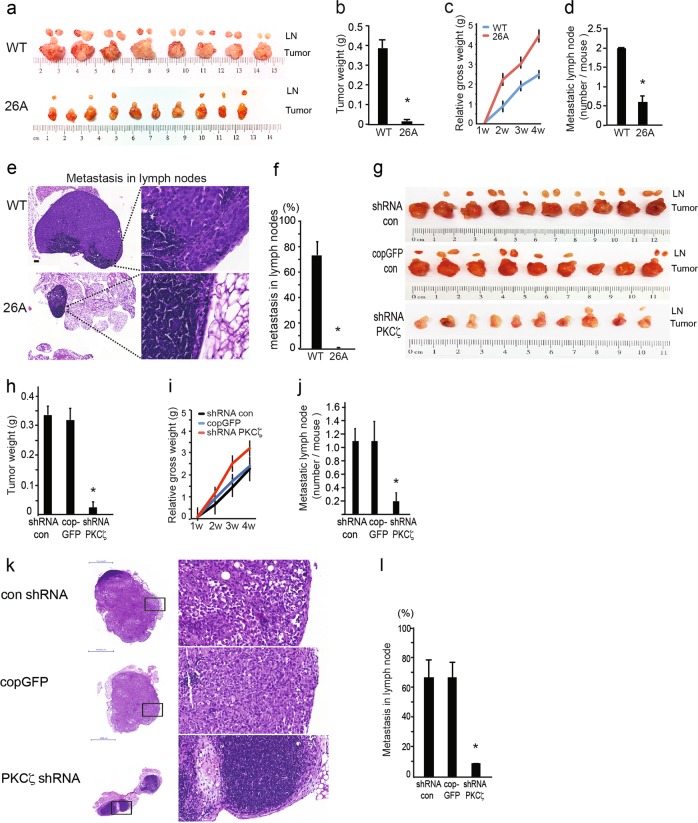
Knockout or knockdown of *PKCζ* inhibits tumor growth and lymphatic metastasis in an orthotopic xenograft mouse model. **a** WT PC3U and PKCζ-knockout 26A cells were injected into the prostate of mice. Tumors and the regional lymph nodes (LNs) were obtained after 4 weeks, WT tumor *n* = 8, LN *n* = 16; 26A tumor *n* = 10, LN *n* = 6. **b** Mean value of tumor weight ± S.E.M. for WT (*n* = 8) and 26A group (*n* = 10), *p ≤ 0.05, Mann–Whitney non-parametric test. **c** Analysis of the gain of body weight in mice with tumor burden by measuring the mouse’s gross weight during tumor growth. Bar graph shows the mean ± S.E.M. from WT (*n* = 8) and 26A (*n* = 10) group. * *p* ≤ 0.05, students’ *t* tests. **d** Quantification of metastatic LNs shows mean ± S.E.M from WT (*n* = 8) and 26A (*n* = 10), *p ≤ 0.05, Mann–Whitney non-parametric test. **e** Representative images of metastatic lymph nodes by Hematoxylin-eosin (H&E) staining. Scale bar, 200 μm. **f** Quantification of the invasive area occupied by cancer cells in the LNs. Bar graph shows the mean ± S.E.M. from WT (*n* = 16) and 26A (*n* = 6), * *p* ≤ 0.05, Mann–Whitney non-parametric test. **g** PC3 cells, stably transfected with control shRNA, cop GFP control, or PKCζ shRNA lentiviral particles, were injected into the prostate of mice, and the tumors and the regional lymph nodes (LNs) were collected after 6 weeks, con shRNA group *n* = 10, LN *n* = 12; cop GFP group: tumor *n* = 9, LN *n* = 10; PKCζ shRNA group *n* = 10, LN *n* = 2. **h** Mean value of tumor weight ± S.E.M. for con shRNA (*n* = 10), cop GFP control (*n* = 9), and PKCζ shRNA group (*n* = 10), * *p* ≤ 0.05, Mann–Whitney non-parametric test. **i** Analysis of the gain of body weight in mice with tumor burden by measuring the mouse’s gross weight during tumor growth. Bar graph shows the mean ± S.E.M. from con shRNA (*n* = 10), cop GFP control (*n* = 9), and PKCζ shRNA group (*n* = 10). **j** Quantification of metastatic LNs shows mean ± S.E.M. from con shRNA (*n* = 10), cop GFP control (*n* = 9), and PKCζ shRNA group (*n* = 10). * *p* ≤ 0.05, Mann–Whitney non-parametric test. **k** Representative images of metastatic lymph nodes by Hematoxylin-eosin (H&E) staining. Scale bar, 1000 μm. **f** Quantification of the invasive area occupied by cancer cells in LNs. Bar graph shows the mean ± S.E.M. from con shRNA (*n* = 12), cop GFP control (*n* = 10), and PKCζ shRNA group (*n* = 2), *p ≤ 0.05, Mann–Whitney non-parametric test

To investigate the role of PKCζ in prostate cancer progression in vivo, we performed animal experiments via orthotopic implantation of WT PC3U cells or *PKCζ*-knockout cells (26A) into the ventral prostate of nude mice. Compared to the WT cells, 26A cells developed into smaller tumors and had less metastasis into adjacent regional lymph nodes (Fig. 5a). The net weight of the formed tumors was significantly reduced in 26A (Fig. 5b), and the mice in the 26A group apparently gained more body weight than the WT group, due to the mouse in the WT group suffered from a larger tumor burden (Fig. 5c). Importantly, markedly fewer metastatic lymph nodes (Fig. 5a, d) and less lymphatic dissemination were detected in mice implanted with the 26A cell clone (Fig. 5a, e, f, g). The metastatic lymph nodes were examined and found to be much larger and more invasive in the WT group than the 26A group (Fig. 5e, f). We extended our study to also compromise PC3 cells, stably transfected with control shRNA, cop GFP control, or PKCζ shRNA lentiviral particles, which were injected in the prostate of mice; and the tumors and the regional lymph nodes (LNs) were collected after 6 weeks. A reduction in tumor size and metastasis in lymph nodes were observed by knock down of PKCζ (Fig. 5g, h), which was reflected in the body weight of the animal (Fig. 5i). Also the number of metastatic lymph nodes and area invaded by cancer cells, was significantly reduced in the *PKCζ* knockdown group (Fig. 5j-l). However, in the mouse xenograft model implanted with LNCaP cells, less metastasis was observed in lymph nodes, even in the WT group (Supplementary Figure 3). This is consistent with our observation in vitro that lower level of PKCζ expression and lower invasiveness in LNCaP cells as shown in Fig. 1. These data are providing evidence for that knocking out *PKCζ* in prostate cancer cells prevented their invasion in vivo. Taken together, our data show that PKCζ may regulate tumor growth and invasion in prostate cancer progression in vivo.

### PKCζ facilitates lymphangiogenesis in mouse xenografts

**Fig. 6:**
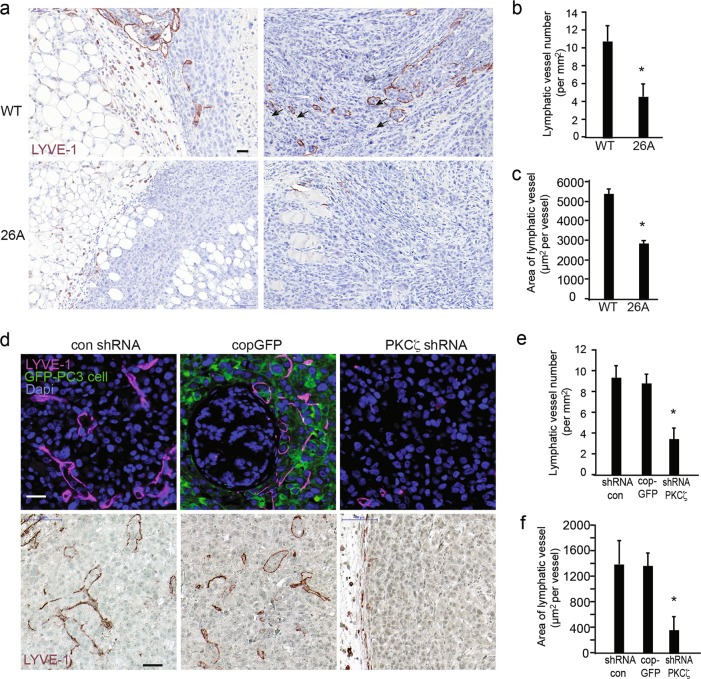
PKCζ facilitates lymphangiogenesis and lymphatic vessel remodeling in mouse xenografts. **a** Representative images of lymphatic vessels in tumors developed from WT and 26A cells immunohistochemically stained for LYVE-1. Scale bar, 50 μm. **b** The number of lymphatic vessels in the images was quantified, given as means ± S.E.M. for 8 samples (8 images) in the WT group and 10 samples (10 images) in the 26A group, *p ≤ 0.05, Mann–Whitney non-parametric test. **c** Analysis of the enlarged lymphatic vessel by quantifying the area of each lymphatic vessel. Values are given as means ± S.E.M. for WT (8 images) and 26A(10 images), *p ≤ 0.05, Mann–Whitney non-parametric test. **d** Representative images of lymphatic vessels in tumors developed from PC3 cells stably transfected with control shRNA, cop GFP control, or PKCζ shRNA lentiviral particles, immunofluorescence staining (upper panel) immunohistochemistry staining (lower panel) and for LYVE-1. Scale bar, 50 μm. **e** The number of lymphatic vessels in the images was quantified, given as means ± S.E.M. from control shRNA (10 images), cop GFP control (9 images), and PKCζ shRNA group (10 images), *p ≤ 0.05, Mann–Whitney non-parametric test. **f** Analysis of the enlarged lymphatic vessel by quantifying the area of each lymphatic vessel. Values are given as means ± S.E.M. from control shRNA (10 images), cop GFP control (9 images), and PKCζ shRNA group (10 images), *p ≤ 0.05, students’ *t* tests. LYVE-1 and GFP antibodies were used at 1:200 dilution

To further elucidate the role of PKCζ in lymphatic metastasis in prostate cancer, we performed immunohistochemistry (IHC) to investigate the lymphatic vessel marker LYVE-1 by staining the tissues dissected from mouse xenografts. Interestingly, a number of lymphatic vessels formed in and around the invasive tumor that developed from WT prostate cancer cells (Fig. 6a), and many vessels were enlarged and irregular, representing the typical remodeling of lymphatic vessels in tumors. However, in the tumors developed from *PKCζ*-knockout cell line 26A, there were fewer lymphatic vessels and less lymphatic vessel remodeling. Quantification of the intratumoral and peritumoral lymphatic vessels revealed significantly different lymphatic vessel density between the WT and 26A groups (Fig. 6b). To verify the enlargement of lymphatic vessels in tumors, the area of each lymphatic vessel was measured and quantified. The size (area) of each lymphatic vessel formed in the WT tumors was significantly larger than the lymphatic vessels formed in the 26A group (Fig. 6c). Notably, the invasion of prostate cancer cells into the enlarged modified lymphatic vessels was detected in the WT group (Fig. 6a), but not the *PKCζ*-knockout group. We next extended our study to involve the effects of PKCζ also on lymphatic vessels in tumors developed from PC3 cells stably transfected with control shRNA, cop GFP control, or PKCζ shRNA lentiviral particles (Fig. 6d–f). Similar effects of knocking down *PKCζ* on the invasiveness of PC3 cells into lymphactic vessels, and the area and size of lymphactic vessels, was observed as in PC3U cells. These data indicate that PKCζ is involved in the regulation of aberrant lymphangiogenesis in prostate cancer progression, and that the intratumoral and peritumoral lymphatics may facilitate the transport of cancer cells to lymph nodes via lymphatic drainage.

### PKCζ is involved in the regulation of aberrant lymphangiogenesis and lymphatic metastatic microenvironement

**Fig. 7:**
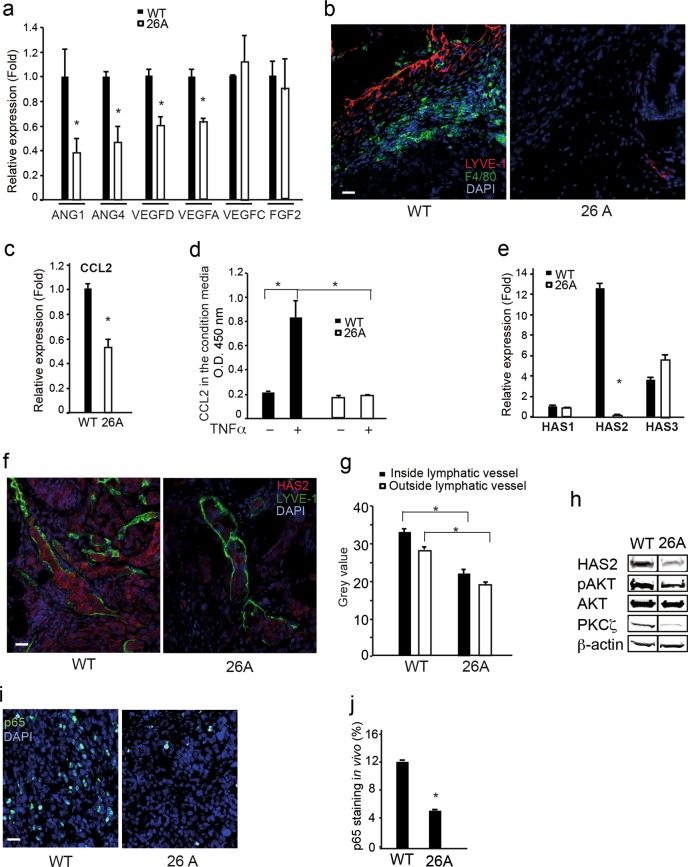
PKCζ is involved in the regulation of aberrant lymphagiogenesis and lymphatic metastatic microenvironment. **a** qRT-PCR analysis for expression of *ANG1*, *ANG4*, *VEGFD*, *VEGFA*, *VEGFC*, and *FGF2* in WT PC3U cells and *PKCζ*-knockout 26A cell clone. *n* = 4 independent experiment, results given as mean ± S.E.M., *p ≤ 0.05, students’ *t* tests. **b** Immunofluorescence staining of lymphatic vessel marker LYVE-1 and macrophage marker F4/80 in tumor tissue from the mouse xenografts model. Scale bar, 50 μm. **c** Analysis of CCL2 expression in WT PC3U cells and 26A cells by qRT-PCR, *n* = 4 independent experiments, results given as mean ± S.E.M., * *p* ≤ 0.05, students’ *t* tests. **d** ELISA analysis for secretion of CCL2 in the media by WT PC3U cells or 26A cells treated with or without TNFα for 6 h, and the results show the mean ± S.D. from 3 independent experiments, *p ≤ 0.05, one-way ANOVA. **e** qRT-PCR analysis for the expression of *HAS1*, *HAS2*, and *HAS3* in WT PC3U cells and *PKCζ*-knockout 26A cells. *n* = 4 independent experiment, results given as mean ± S.E.M., *p ≤ 0.05, students’ *t* tests. **f** Immunofluorescence analysis for the localization of HAS2 inside or outside lymphatic vessels (marker: LYVE-1) in WT tumors or 26A tumors from the mouse xenografts model. Scale bar, 50 μm. **g** Quantification of the intensity of HAS2 staining inside or outside lymphatic vessels from WT tumors (*n* = 8 images) and 26A tumors (*n* = 10 images) by ImageJ software, given as mean ± S.E.M., *p ≤ 0.05, Mann–Whitney non-parametric test. **h** Western blot analysis of HAS2, pAKT, PKCζ expression in tumor tissue in the mouse xenografts model. **i** Immunofluorescence staining of p65 in tumor tissue developed from WT PC3U cells or 26A cells in the mouse xenografts model. **j** Quantification of p65-staining in the nuclei (co-localization with DAPI) among 300 cells in the image of tumor tissue, WT (*n* = 8 images); 26A (*n* = 10 images), values were given as mean ± S.E.M., *p ≤ 0.05, Mann–Whitney non-parametric test. Dilutions for antibodies used in immunofluorescence; LYVE-1 (1:200), F4/80 (1:50), HAS2 and p65 (1:100). Dilutions for antibodies used in western blotting: HAS2 (1:200), pAKT, AKT, PKCζ and β-actin (1:1000)

It is of interest to understand how PKCζ regulates aberrant lymphangiogenesis during tumor progression. Therefore, we investigated the expression of lymphangiogenic factors by performing qRT-PCR of WT PC3U and PKCζ-knockout cells (clone 26A). Interestingly, the expression of VEGF-A, VEGF-D, ANG1, and ANG4 in WT PC3U cells was apparently higher than in the 26A cell clone, whereas no differential expression was found for VEGF-C and FGF2 (Fig. 7a). Monocytes/macrophages are recruited to tumor tissue, and these inflammatory cells are playing an important role in angiogenesis and lymphangiogenesis during tumor progression [26]. Therefore, we further investigated the recruitment of monocytes/macrophages in tumor tissues by immunostaining with mouse macrophage marker F4/80. Remarkably, the number of monocytes/macrophages recruited to the tumors that were initiated from WT PC3U cells was high, and only a few monocytes/macrophages were detected in the tumors initiated from the 26A cell clone (Fig. 7b).

We also investigated the chemotactic cytokine C-C motif chemokine ligand 2 (CCL2), referred to as monocyte chemoattractant protein 1 (MCP1), which is important for the recruitment of monocytes/macrophages during tumor metastasis [27]. The expression of CCL2 was inhibited when *PKCζ* was knocked out in the 26A cell clone (Fig. 7c). CCL2 secreted into the culture media from WT and 26A cells was measured by ELISA. Interestingly, the levels of secreted CCL2 in the supernatant from WT PC3U cells was dramatically increased upon TNFα stimulation, but no such response was observed in the 26A cell clone (Fig. 7d). These data suggest that PKCζ regulates the expression of lymphangiogenic factors in prostate cancer cells and the expression of CCL2, which is important for recruitment of macrophages and subsequent lymphangiogenesis in cancer progression and dissemination.

HA is a multifunctional molecule in the extracellular matrix and can interact with the cell surface receptor LYVE-1, which is expressed in the lymphatic vessels [21]. HA is synthesized by HASs (HAS1, HAS2, and HAS3). Lymphatic vessels drain a large number of HA molecules via LYVE-1; therefore, we investigated the expression of HASs. Interestingly, expression of HAS2 was apparently higher in the WT PC3U cells than in the 26A cell clone. We performed immunoblotting to detect the protein levels in tumors that developed from mouse xenografts. HAS2 was highly expressed in WT tumors but down-regulated in the tumors that developed from 26A cells (Fig. 7e). Co-immunofluorescent staining for HAS2 and LYVE-1 showed that a portion of prostate cancer cells that localized in the lymphatic vessels expressed dramatically higher levels of HAS2, with significant differences in the level of HAS2 in prostate cancer cells outside or inside the lymphatic vessels (Fig. 7f, g). Similar results were also obtained in PC3 tumors in the mouse xenograft model (Supplementary Figure 3). We also observed that the tumors that developed from the 26A cell clone had decreased AKT signaling (Fig. 7h), as expected and in line with our in vitro data presented in Fig. 2d. This indicates that more aggressive prostate cancer cells invade the lymphatic vessels, as they expressed more HAS2. As NF-κB signaling is known to regulate the expression of HAS2 [19], we detected activated p65 in the tumor tissue from xenografts by immunofluorescence. There was apparently more p65 in the nuclei of WT tumors, and clearly less nuclear p65 in the tumor tissues derived from the 26A cell clone (Fig. 7i, j). Taken together, these data indicate that PKCζ is important for the activation of NF-κB, which then regulates the expression of HAS2 and facilitates HA production and cancer cell dissemination via lymphatic drainage.

## Discussion

In this study, we have investigated the correlation between expression of PKCζ and aggressivness of prostate cancer cells. As reported here, we observe that high levels of PKCζ is regulating growth factor induced activation of AKT in response to TGFβ, and activation of AKT and NFκB in response to EGF and TNFα, which also can explain their aggressiveness due to higher capability to proliferate and be invasive in vitro. The recent development of innovative genome editing technologies, such as ZFNs, TALENs, and CRISPR/Cas9, have shown the power of successful genetic manipulation, both in cell lines and human disease models [22–24]. By using ZFN technology or lentiviral short hairpin siRNA particles, the *PKCζ* gene was specifically knocked out or knocked down from the aggressive castration-resistant human prostate cancer cell line PC3U and PC3 cells. This allowed us to study in detail, the molecular mechanisms whereby PKCζ modulates lymphatic metastasis of prostate cancer cells in vivo.

The role of PKCζ in prostate cancer progression is complicated, as it exerts multiple functions in the regulation of different cell events temporally and spatially. Kim et al. reported that PKCζ suppresses prostate tumorigenesis in the early stage of neoplasia in a Pten^+/-^ mouse model [28]. However, PKCζ promotes the aggressive phenotype of human prostate cancer cells [5] and facilitates the migration and invasion of prostate cancer cells via formation of the Par6-PKCζ complex in the leading edge of membrane ruffles [14]. These studies suggest that PKCζ likely has an oncogenic role that facilitates cancer cell invasion and metastasis in the later stages of prostate cancer progression. Here, we used a traditional xenograft model and found that *PKCζ*-knockout or knockdown from PC3U and PC3 cells prevented their invasive growth and lymphatic metastasis in mouse orthotopic xenografts. We found that this occurred due to *PKCζ*-knockout impairing angiogenesis and lymphangiogenesis during tumor development.

Interestingly, we found that PKCζ regulates prostate cancer cell secretion of substantive amounts of CCL2 and promote TAM recruitment. CCL2 was recently found to be a diagnostic, predictive, and prognostic biomarker of prostate cancer [29] and has been reported to be the most important chemokine for monocyte recruitment to prostate tumors [17, 30]. Moreover, we also observed that PKCζ knockout remarkably impaired the secretion of CCL2 by prostate cancer cells and less infiltration of monocyte/macrophages into the tumors initiated from the 26A cell clone (Fig. 7b). Macrophages play a complex role in cancer depending on their phenotype, either promoting or inhibiting cancer progression [31]. In prostate cancer, the M2 macrophages are typically a “pro-tumor” phenotype, and the presence of tumor-associated M2 macrophages strongly correlates with angiogenesis, metastases and poorer patient outcomes [32]. In our orthotopic xenografts derived from the WT prostate cancer-initiated tumors, we found a number of macrophages in the invasive zone, which is important for continuous release of chemokines and lymphatic growth factors to facilitate tumor metastasis (Fig. 7b).

Both tumor cells and tumor-associated macrophages express lymphangiogenic factors [33]. VEGFC/D and VEGFR3 are the key pathways for lymphangiogenesis;[34] other important molecules include VEGFA and VEGFR2, angiopoietins ANG1, ANG2, ANG4, and TIE2, FGF2, BMP9, ALK1, Notch1, and ephrin B2 [35]. Interestingly, we found that PKCζ regulates the expression of *VEGFD*, *VEGFA*, *ANG1*, and *ANG4* in prostate cancer cells (Fig. 7a), which may facilitate the generation of aberrant lymphangiogenesis in tumors. However, the detailed mechanism, including which signaling pathway is involved in regulating the expression of lymphangiogenic factors via PKCζ, is still unknown and needs to be further investigated. Some studies have reported that lymphatic density is an accurate independent predictor of poor survival and increased metastatic potential in human tumors [36–40]. Our study showed that *PKCζ* knockout impaired lymphangiogenesis in two orthotopic prostate cancer models; both PC3U and PC3, and that the lymphatic density was remarkably lower in tumors initiated from *PKCζ*-knockout cell clones, which correlated with less lymphatic metastasis in the in vivo model.

NF-κB signaling is constitutively active in aggressive prostate cancer, playing a central role in modulating tumor stroma [41]. The activation of NF-κB signaling controls the induction of cytokines and matrix metalloproteinases in tumor stroma, mediating tumor angiogenesis and metastasis [42, 43]. Vigetti et al. reported that NF-κB also regulates the induction of HA in human endothelial cells [19]. Interestingly, we found that PKCζ is involved in the activation of NF-κB signaling in human prostate cancer xenografts, and that PKCζ regulates the expression of HAS2; thus, PKCζ might indirectly manipulate the amount of HA in the extracellular matrix, a factor that promotes prostate tumor growth and is associated with a poor prognosis [20]. HA mediates lymphatic drainage by binding LYVE-1. Through passive lymphatic drainage, cancer cells disseminate to the sentinel lymph nodes or distal organs. It was originally thought that tumors invade lymphatic vessels via passive drainage. However, recent data indicate a more complex and specific mechanism behind lymphatic metastasis. Lymphatic endothelial cells have been reported to highly express CCL21, whereas its receptor CCR7 is highly expressed in breast cancer cells and melanoma cells and predicted to be the mechanism by which breast cancer and melanoma preferentially metastasize to lymph nodes [33, 44]. However, if there is a specific interaction between prostate cancer cells and the lymphatic endothelium, or if other molecules specifically mediate this interaction still needs to be investigated. Our study proposes that PKCζ maybe represents a novel drug target in prostate cancer to prevent tumor progression and lymph node metastasis.

## Materials and methods

### Cell culture

The human prostate cancer cell line PC3 and human lung cancer cell line A549 were purchased from ECACC (Salisbury, UK). The human prostate cancer cell line LNCaP were purchased from ATCC (Manassas, USA). PC3U cells were originally derived from PC3 cells and express more TGFβ receptors than PC3 [45]. The cancer cell lines were grown in RPMI-1640 with 10% fetal bovine serum (FBS) and L-glutamine (Sigma, St. Louis, MO, USA). The human immortalized normal prostate cell line RPWE1 were from ATCC (Manassas, USA) and cultured in the Keratinocyte Serum-Free Medium with bovine pituitary and human recombinant epidermal growth factor (Gibco-Thermo Scientific, Waltham, MA, USA). The cells were starved 12–18 h in medium supplemented with 1% FBS before EGF, TGFβ (Prospec, Ness-Ziona, Israel), and TNFα (Gibco-Thermo Scientific) stimulation at a concentration of 10 ng/ml. The cell lines were routinely tested for mycoplasma contamination and a panel of pathogens, following the guidelines of Umeå University Animal Care Committee.

### Antibodies and reagents

The antibodies against PKCζ, pAKT, AKT, TRAF6, APPL1, pERK, ERK, pSmad2, Smad2, P65, LaminA, were purchased from Cell Signaling Technology, Inc. (Beverly, MA, USA) and used at 1:1000 dilution for Western blotting. HAS2 (Santa Cruz, CA, USA) was used at 1:200, and β-actin and β-tubulin (Sigma, St. Louis, MO, USA) was used at 1:1000 for Western blotting. Antibodies against LYVE-1, GFP, F4/80 (Abcam, UK), Ki67 was used at 1:500 dilution (Dako, Denmark), Alexafluor 488 and Alexafluor 555 (Invitrogen, Oregon, USA) were used for immune-staining. 4,6-Diamidino-2-phenylindole dihydrochloride (DAPI) was from Merck, (Darmstadt, Germany). Pefabloc was from Roche (Mannheim,Germany), and PageRuler prestained protein ladder was from Thermo Scientific.

### Western blotting

The cultured cells was washed once with ice-cold PBS and lysed in ice-cold lysis buffer (150 mM NaCl, 50 mM Tris pH 8.0, 0.5% (v/v) DOC, 1% (v/v) NP40, 10% (v/v) glycerol, 1 mM aprotinin, 1 mM Pefabloc and 2 mM sodium orthovanadate). The tumor tissue from mouse experiments was lysed in the above lysis buffer with Tissuelyser II (Qiagen, Germany). After centrifugation, the supernatants were collected and protein concentrations were determined by BCA protein measurement kit (Thermo Scientific). Equal amount of protein from total cell lysate was run in SDS-PAGE and transfered on to polyvinylidine difluoride membranes. The membranes were blocked for 1 h with 5% BSA in TBS-T and then incubated with specific antibodies overnight at 4 °C. Then the membranes were washed 3 times with TBS-T, incubated for 1 h with a secondary horseradish peroxidase-conjugated antibody and developed with ECLWestern blotting detection reagent and detected with Amersham Imager RBG system (GE Healthcare, Buckinghamshire, UK).

### XCELLigence real-time cell proliferation assay

Experiments were carried out using the RTCA DP xCELLigence Analyzer and E16 xCELLigence plate (ACEA Biosciences, Inc. San Diego, CA, USA). The E16 plates were prepared by addition of complete media (50 μl) to each well. After equilibration to 37 °C, plates were inserted into the xCELLigence, and the base-line impedance was measured to ensure the wells and connections are working. Then 20000 freshly split cells in 50 μl media were added into each well, and the xCELLigence system start to detect changes in impedance over time, every 5 min in the first 5 h, and then every 15 min in 72 h. Inhibitor was added into the cells 1 h after seeding. Data was calculated using RTCA software 2.0, supplied with the instrument.

### Invasion assay

Invasion assay was performed with the CytoSelect™ Cell Invasion Assay kit (Cell Biolabs, Inc., San Diego, CA). The collagen layer of the cell culture inserts were rehydrated in 300 μL serum-free RPMI-1640 media, and 1 × 10^6^ cells were seeded into the upper side of the chambers in 1% serum RPMI-1640. Lower wells of the invasion plates were filled with 500 μl RPMI with 10% FBS. Non-invasive cells were removed from the upper chamber after growing for 24 h, and the invasive cells were photographed with a Leica DMR light microscope after staining with crystal violet cell stain solution. Colorimetric quantification was performed by transfer inserts into 200 μL of extraction solution for 10 min. Optical density (O.D.) at 560 nm was measured in a 96-well plate by Multiscan FC Microplate Photometer (Thermo Scientific).

### Generation of PKCζ-deficient cell line

CompoZr knockout Zinc Finger Nucleases (ZFN) Kit designed for *PKCζ*-knockout was purchased from Sigma-Aldrich (St. Louis, MO, USA). According to the manufacturers protocol, PC3U cells were seeded in a 6-well plate 1 day before, and then two ZFN plasmids were transfected into the cells at 50% confluence with Fugene 6 (Promega, Madison WI, USA). After transfection for 2 days, part of cells were harvested for DNA analysis, and part of cells were diluted for a single cell culture in a 96-well plate until single clones were obtained. These cell clones were cultured and stored at –160 °C for further confirmation.

### shRNA lentiviral particles transduction and selection of the stable clones

PKCζ shRNA lentiviral particles, control shRNA lentiviral particles, and copGFP control lentiviral particles, were purchased from Santa Cruz Biotechnology (Texas, USA). Following the manufacturers instruction, the target cells were seeded in a 12-well plate for 24 h before the transfection, and became approximately 50% confluent on the day of infection. After the media was replaced by the transfection media with Polybrene (Santa Cruz) at a concentration of 5 μg/ml, the shRNA lentiviral particles were added into the cells. The transfection media was removed after 24 h, and then the cells were cultured in complete media for 48 h. To select the stable clones expressing the shRNA, the infected cells were cultured in the media with Puromycin at 5 μg/ml for 2 weeks, and then several colonies were picked up and expanded for validation of knock down of endogenous PKCζ.

### PCR and Single-strand conformation polymorphism (SSCP) analysis

Genomic DNA was exacted from cell lines with the Genomic DNA Miniprep Kit (Sigma-Aldrich). ZFN cutting site was amplified with forward primer 5′–3′AATATGCCCCACGGTAACA, reverse primer 5′–3′ ATAAGCATCTGTGGCCAACC for PCR amplification (95 °C for 5 min, 30 cycles from 95 °C for 30s, 57 °C for 30 s to 72 °C for 30 s, final extension for 7 min at 72 °C, hold at 4 °C). PCR products were mixed with equal volume of SSCP loading buffer (0.05% bromophenol blue, 0.05% xylene cyanol, 95% formamide, 10mM EDTA), denatured at 95 °C for 5 min, rapidly cooled on ice, and then loaded on a precast 12.5% acrylamide gel.

### Cel-1 assay or Surveyor nuclease assay

A total of 10 µl of PCR product from above was used in a slow temperature decreasing program: 95 °C for 10 min, decreasing the temperature form 95 °C to 85 °C by –2.0 °C/s, keep the temperature at 85 °C for 1 min, decreasing the temperature from 85 to 25 °C by –0.3 °C/s, and hold at 4 °C. Thereafter, SURVEYOR Nuclease (Transgenomic, Omaha, USA) was added to the denatured and re-annealed PCR products to incubate for 2 h, and then the digestion products were separated on the 10% polyacrylamide gel in 1× TBE buffer, and visualized by silver staining method.

### Sequencing analysis

Genomic DNA from Cel-1 assay positive cells was purified and ZFN cutting site was PCR amplified as described above. Sequencing PCR (BigDye Teminator v 3.1 cycle sequencing kit) was performed using this amplified PCR product. The sequencing results were analyzed on Sequence Scanner v 1.0 software.

### Immunofluorescence staining for cultured cells

Cells were grown on the sterile glass microscopy slides in a 6-well plate under the indicated condition. The slides were washed 4 times with PBS, fixed in 4% paraformaldehyde for 30 min at room temperature, washed 4 times in PBS and subsequently permeabilized in 0.2% Triton X-100 in PBS for 10 min, and washed again 4 times in PBS, blocked in 10 mM glycine overnight. Then, the slides were incubated with primary antibody anti-Ki67 (1:500) in a humid chamber for 1 h at room temperature. After washing with PBS, the slides were incubated with fluorescent dye labeled second antibodies for 45 min hours at room temperature, and then with DAPI for 5 min. Thereafter, the mount medium was added on the slides for imaging. The samples were analyzed in a fluorescence microscope (Axioplan 2; Carl Zeiss Microimaging, Inc.) with a digital camera (C4742–95; Hamamatsu), using a Plan-neofluar ×63 objective lens (Carl Zeiss MicroImaging, Inc.); photography was performed at room temperature. Primary images were acquired with the camra’s QED software.

### Xenografts animal experiment

All mouse experiments were carried out in strict accordance with the guidelines of Umeå University Animal Care Committee, and under the conditions and procedures approved by the Umeå Ethical Animal Review Board (ID: A19–13, A7–18). Five- to 6-weeks old male athymic nude mice (Hsd:Athymic Nude- Foxn1^nu^, Harlan) were purchased from Harlan laboratories, UK, and maintained at the animal facility at Umeå University. For the orthotopic injection, 2% isoflurane were used to anesthetize the mice and the prostates were exposed with an abdominal incision, and then 1x10^5^ PC3U cells, 3 × 10^5^ PC3, or 3x10^5^ LNCaP cells were injected into the prostate. In total 80 mice were used in this study, with ten mice randomly included in each group. The mice were monitored and body weight was measured twice a week. There were totally 5 mice dead before the planned sacrifice, therefore, they were not included in the analysis. After injection of PC3U cells for 4 weeks, or PC3 or LNCaP cells for 6 weeks, the animals were sacrificed. Tumors and regional lymph nodes were resected, and weighted. Small portions of tumor tissues were froozen in liquid nitrogen for protein and RNA extraction. The remaining tumor tissues and lymph nodes were fixed in formalin and embedded in paraffin for morphology evaluation with light microscopy.

### Immunohistochemistry staining for paraffin-embedded sections

Paraffin-embedded sections were rehydrated in xylene 2 times each for 10 min, 100% ethanol for 10 min, 95%, 80%, 70% ethanol and deionized H_2_O each for 5 min, and then in PBS for 10 min. Thereafter the sections were treated with Antigen Retrieval Reagent (R&D System, MN, USA) at 95 °C for 5 min, rinsed with PBS. Then the sections were kept in 0.75% H_2_O_2_/75% methanol for 30 min, washed twice with PBS, and blocket in 5% normal goat serum for 1 h at room temperature. The sections were incubated with primary antibodies overnight at 4 °C, LYVE-1 (1:200). After being washed with PBS 3 times each for 5 min, the sections incubated with secondary antibody (DAKO envision system, Denmark) for 45 min at room temperature followed by three washes in PBS. Then, the sections were developed with AEC (Vector Laboratories, Burlingame, CA, USA), counterstained with hematoxylin and mounted in aqueous mounting medium (Vector Laboratories). Digital images for IHC were acquired by scanning with Pannoramic 250 Flash II (3DHistech, Hungary), and then examined by two pathologists at Umeå University Hospital, and analyzed by ImageJ software.

### Immunofluorescence staining for paraffin-embedded sections

Sections from animal experiments were deparaffinized and retrieved as described above, and thereafter blocked in 1% horse serum in PBS for 1 h at room temperature, and then incubated with primary antibodies overnight at 4 °C, LYVE-1 (1:200), GFP (1:200), F4/80 (1:50), HAS2 (1:100), p65 (1:100). After being washed with PBS 3 times each for 5 min, the sections were incubated with NorthernLights secondary antibody (R&D) for 45 min at room temperature. Then the sections were washed with PBS 3 times each for 5 min, mounted with mounting media with DAPI and visualized with a fluorescence microscope (Axioplan 2; Carl Zeiss Microimaging, Inc.).

### Quantitative assessment

For the digital images, a semi-automated algorithm was used to extract detailed morphometric data on the diameter, area, numbers of vessels, and distribution of different sizes of vessels by using modified software (Image J, National Institutes of Health, Bethesda, MD). Finally the total area and volume was summed up for each tumor. The data was expressed a vascular segment numbers, representing the total numbers of vessels of specified diameters, and counted in all reformatted cross-sections. The intensity of HAS2 were also measured by ImageJ software, and mean pixel intensity values (Gray-scale values) were calculated to compare the intensity of staining.

### Conditioned medium preparation and ELISA

WT and 26A PC3U cells were cultured with or without TNFα stimulation for 6 h. The conditioned medium was collected, centrifuged at 300 × *g* for 10 min, and then centrifuged with Ultracel-3K Centrifugal filters (Merck Millipore Ltd, Tullagreen Carrigtwohill Co, Ireland) at 4000 × *g* for 30 min. Secretion of CCL2 into the medium was determined by using the DuoSet ELISA -Human CCL2/MCP-1 kit (R&D Systems, Minneapolis, MN, USA), according to the manufacturer’s protocols. Optical density (O.D.) at 450 nm was measured in a 96-well plate by Multiscan FC Microplate Photometer (Thermo Scientific).

### Whole exome sequencing (WES) and data analysis

Genome DNA was purified with Allprep DNA/RNA/protein mini kit (Qiagen, Cat.80004), subjected to WES at Novogene (Novogene Bioinformatics Institute, Beijing, China). Single nucleotide polymorphisms (SNPs) and small insertions/deletions (indels) were identified using GATK HaplotypeCaller (version 4.0).

### RNA preparation and qRT-PCR

RNA from WT and 26A PC3U cells was extracted using AllPrep DNA/RNA/Protein Mini Kit (QIAGEN, Cat. 80004) following manufacturer’s instructions. Purified total RNA (2 μg) was used as a template for cDNA synthesis with Thermoscript RT-PCR system (Invitrogen Cat.11146016) according to the manufacturer’s instruction. Purified cDNA was amplified and measured with Stratagene RT-PCR system, and SYBR Green (Applied Biosystems Cat.4385612) was used for detection of PCR products. The following primers (5′–3′) were used for qRT-PCR.

ANG1 FP GGGCCCAGTTACTCCTTACG

ANG1 RP TGTGAACTCAAACGGCTCCA

ANG4 FP TGTGACATGGAGGGTGTTCG

ANG4 RP CCCACCATTGGTCATGGGAA

VEGFA FP AGGAGGGCAGAATCATCACG

VEGFA RP CTGGAAGATGTCCACCAGGG

VEGFC FP TGGGGAAGGAGTTTGGAGTC

VEGFC RP GTTACTGGTTTGGGGCCTTG

VEGFD FP TCTGAACAGCAGATCAGGGC

VEGFD RP AACCTAGTGGACCGATGGGA

FGF2 FP TCTATGTCGTGGAAGCACCG

FGF2 RP GAAGGGTCTCCCGCATACTC

CCL2 FP TCTCAAACTGAAGCTCGCACT

CCL2 RP GGGAATGAAGGTGGCTGCTA

HAS1 FP GAGCCTCTTCGCGTACCTG

HAS1 RP CCTCCTGGTAGGCGGAGAT

HAS2 FP CTCTTTTGGACTGTATGGTGCC

HAS2 RP AGGGTAGGTTAGCCTTTTCACA

HAS3 FP CAGCCTATGTGACGGGCTAC

HAS3 RP CCTCCTGGTATGCGGCAAT

### Statistical analysis

Statistical analysis were performed with a two-tailed unpaired Student’s *t* test for two groups comparision or one-way ANOVA for multiple comparision to determine if treatments groups were significantly different from each other. The non-parametric Mann–Whitney *U* test was used in vivo data. *p* ≤ 0.05 was considered as statistically significant. Error bars represent standard deviation of the mean, unless otherwise is indicated. Statistical tests and the number of repeats are described in the figure legends.

## Supplementary information

Supplementary material text file

Supplementary Figure 1

Supplementary Figure 2

Supplementary Figure 3

Supplementary Figure 4
