# Supplementary material for: PKCζ facilitates lymphatic metastatic spread of prostate cancer cells in a mice xenograft model
Source: Oncogene. 2019 Jan 31;38(22):4215–31. doi: 10.1038/s41388-019-0722-9 (PMC6756056; doi:10.1038/s41388-019-0722-9)
Supplement: Supplementary file 1 — Supplementary material text file [file 41388_2019_722_MOESM1_ESM.docx]

**Supplementary Information ONC-2017-02289R**

**Supplementary Figure 1.** The whole genome sequencing for PC3 and PC3U cells were performed and the gene mutations were detected. The difference between PC3 and PC3U cells was shown as the number of gene mutations (**A**), and percentage of mutant genes (**B**).

**Supplementary Figure 2. PKCζ pseudosubstrate inhibits activation of p65.** Representative images of p65 by immunofluorescence staining in PC3 cells treated with or without PKCζ pseudosubstrate upon TNFα stimulation for 6 hours. Dilutions for antibodies used in immunofluorescence; p65 (1:100).

**Supplementary Figure 3. Knockdown of PKCζ in LNCaP cells doesn’t affect tumor growth and lymphatic metastasis in the orthotopic xenograft mouse model.** (**A**) LNCaP cells, WT or stably transfected with control shRNA, or PKCζ shRNA lentiviral particles, were injected in the prostate of mice, and the tumors and the regional lymph nodes (LNs) were collected after 6 weeks, WT group: tumor n=9, LN n= 5; con shRNA group n=9, LN n=2; PKCζ shRNA group n=10, LN n=1. (**B**) Mean value of tumor weight ± S.E.M. for WT (n=9), con shRNA (n=9), and PKCζ shRNA group (n=10). (**C**) Quantification of metastatic LNs shows mean± S.E.M. from WT (n=5), con shRNA (n=2), and PKCζ shRNA group (n=1).

**Supplementary Figure 4. Knockdown of PKCζ in PC3 cells induces lower level of HAS2 in the tumors in the orthotopic xenograft mouse model.** (**A**) Immunofluorescence analysis for the localization of HAS2 inside or outside lymphatic vessels (marker: LYVE-1) in tumors developed from PC3 cells stably transfected with control shRNA, cop GFP control, or PKCζ shRNA lentiviral particles. Scale bar, 50 μm. (**B**) Quantification of the intensity of HAS2 staining inside or outside lymphatic vessels from control shRNA (10 images), cop GFP control (9 images), and PKCζ shRNA group (10 images) by ImageJ software, given as mean ± S.E.M., * *p* ≤ 0.05, students’ *t* test. Dilutions for antibodies used in immunofluorescence; LYVE-1 (1:200) and HAS2 (1:100).
