# Supplementary figures and images for: PKCζ facilitates lymphatic metastatic spread of prostate cancer cells in a mice xenograft model
Source: Oncogene. 2019 Jan 31;38(22):4215–31. doi: 10.1038/s41388-019-0722-9 (PMC6756056; doi:10.1038/s41388-019-0722-9)

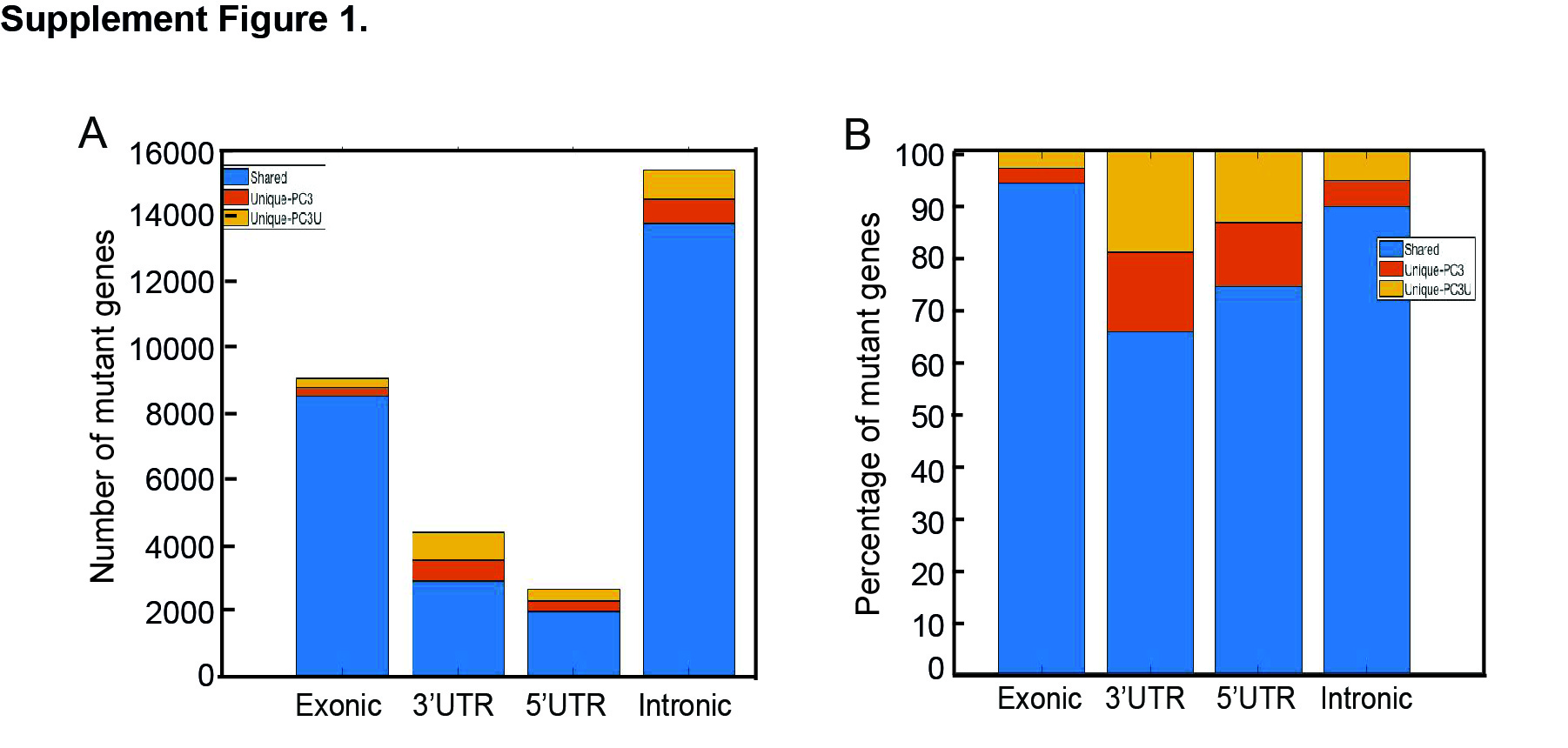

Supplement: Supplementary file 2 — Supplementary Figure 1 [file 41388_2019_722_MOESM2_ESM.jpg]

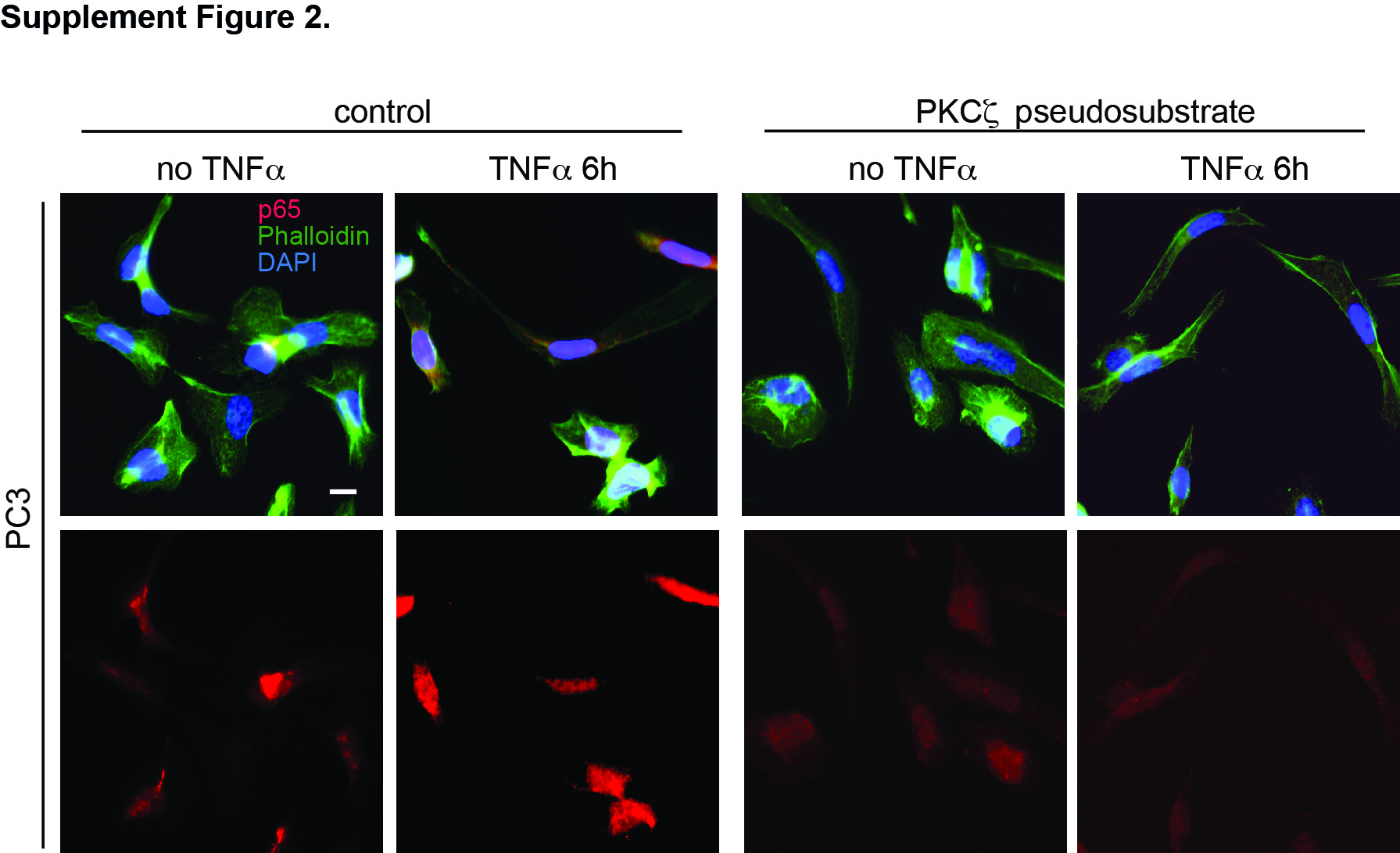

Supplement: Supplementary file 3 — Supplementary Figure 2 [file 41388_2019_722_MOESM3_ESM.jpg]

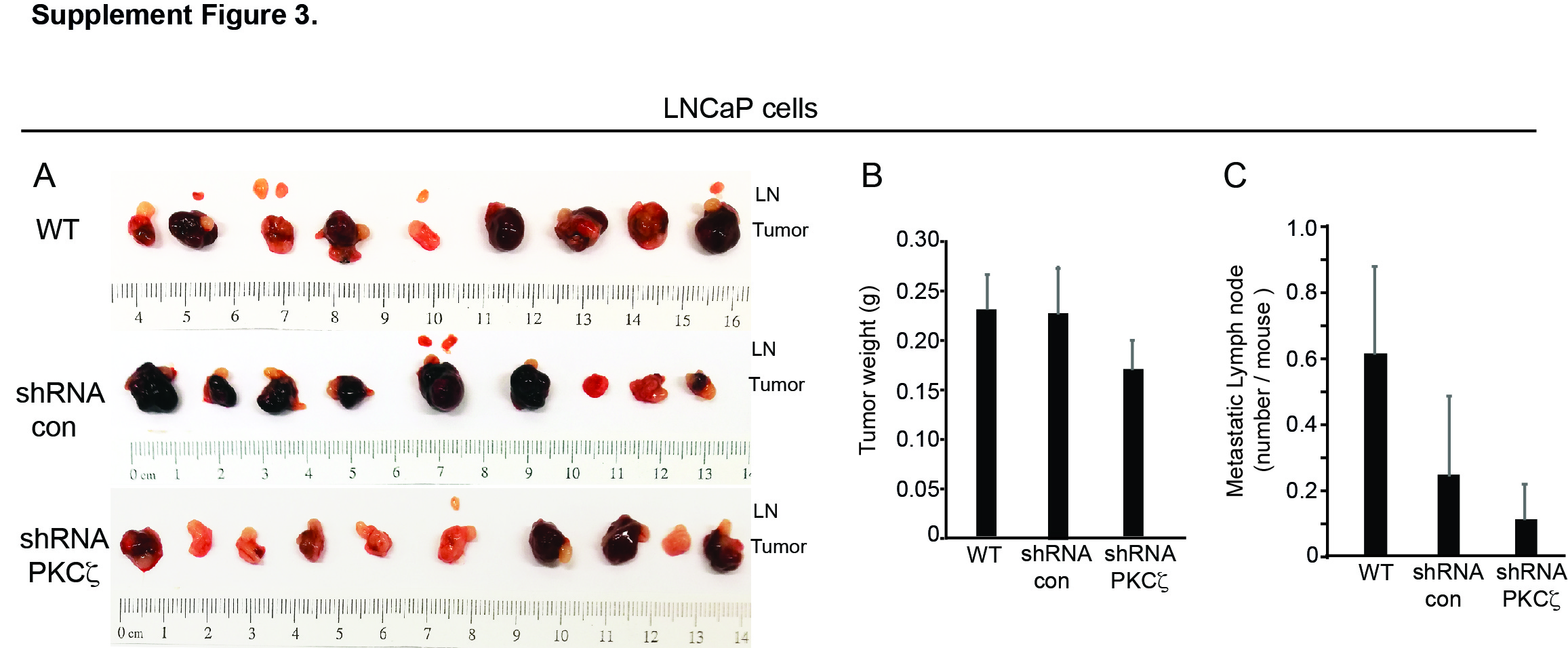

Supplement: Supplementary file 4 — Supplementary Figure 3 [file 41388_2019_722_MOESM4_ESM.jpg]

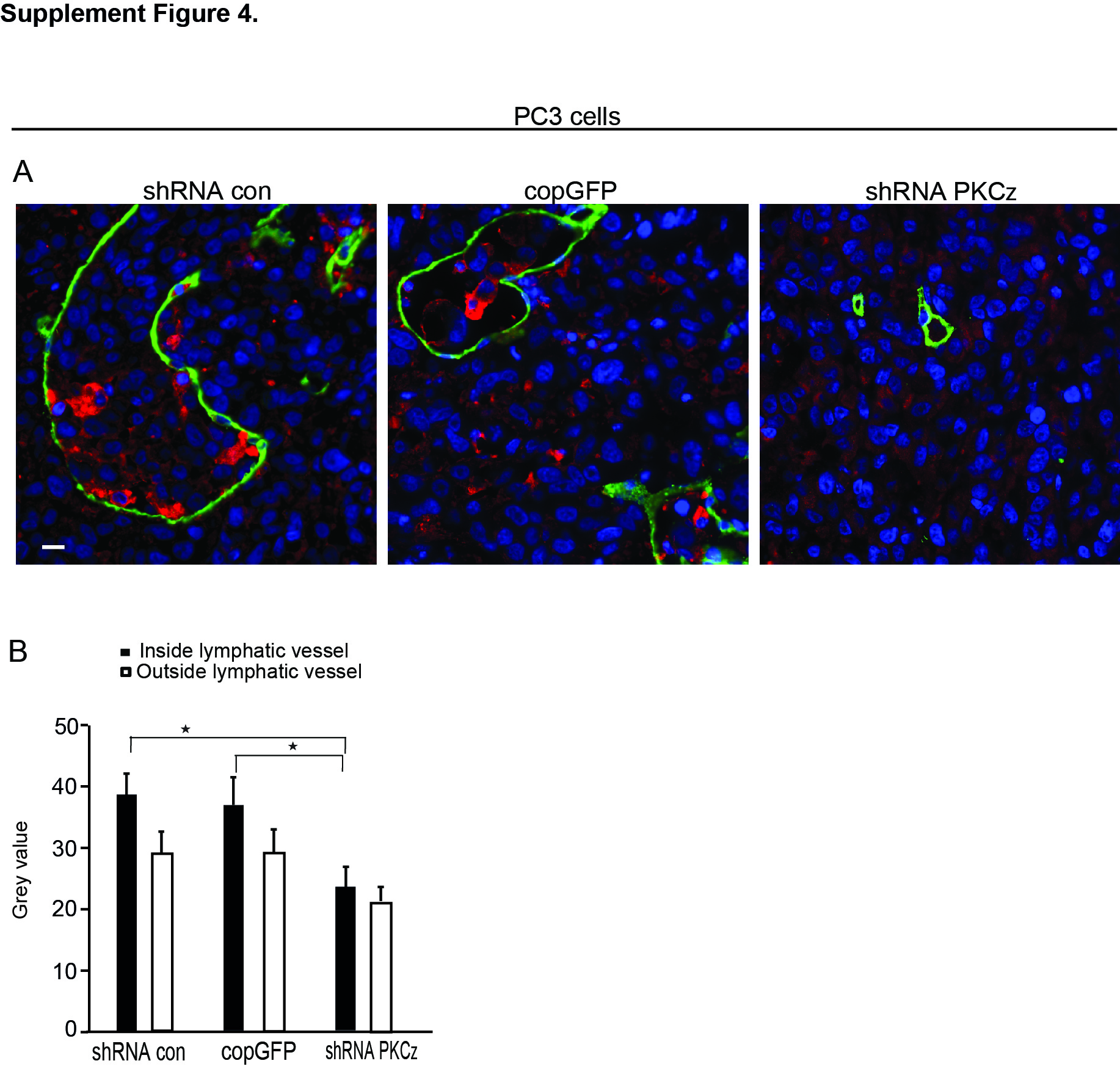

Supplement: Supplementary file 5 — Supplementary Figure 4 [file 41388_2019_722_MOESM5_ESM.jpg]
